# *Brd1* Gene in Maize Encodes a Brassinosteroid C-6 Oxidase

**DOI:** 10.1371/journal.pone.0030798

**Published:** 2012-01-26

**Authors:** Irina Makarevitch, Addie Thompson, Gary J. Muehlbauer, Nathan M. Springer

**Affiliations:** 1 Department of Biology, Hamline University, Saint Paul, Minnesota, United States of America; 2 Department of Agronomy and Plant Genetics, University of Minnesota, Saint Paul, Minnesota, United States of America; 3 Microbial and Plant Genomics Institute, University of Minnesota, Saint Paul, Minnesota, United States of America; 4 Department of Plant Biology, University of Minnesota, Saint Paul, Minnesota, United States of America; University of New England, Australia

## Abstract

The role of brassinosteroids in plant growth and development has been well-characterized in a number of plant species. However, very little is known about the role of brassinosteroids in maize. Map-based cloning of a severe dwarf mutant in maize revealed a nonsense mutation in an ortholog of a brassinosteroid C-6 oxidase, termed *brd1*, the gene encoding the enzyme that catalyzes the final steps of brassinosteroid synthesis. Homozygous *brd1–m1* maize plants have essentially no internode elongation and exhibit no etiolation response when germinated in the dark. These phenotypes could be rescued by exogenous application of brassinolide, confirming the molecular defect in the maize *brd1-m1* mutant. The *brd1-m1* mutant plants also display alterations in leaf and floral morphology. The meristem is not altered in size but there is evidence for differences in the cellular structure of several tissues. The isolation of a maize mutant defective in brassinosteroid synthesis will provide opportunities for the analysis of the role of brassinosteroids in this important crop system.

## Introduction

Manipulation of plant height and growth habits to adjust plant architecture may allow for improved agronomic production in crop plants, from biofuel applications to more efficient use of available resources [1]. Gibberellins (GAs), a large group of cyclic diterpene compounds that promote stem elongation, and brassinosteroids (BRs), commonly occurring steroid hormones that regulate multiple aspects of plant growth and development, are two classes of hormones that alter plant architecture when aberrations occur in their biosynthesis or signaling pathways [1], [2]. Mutations in GA-related genes are responsible for the semi-dwarf phenotypes associated with the green revolution [3] and dwarf or semi-dwarf phenotypes have potential as targets for further agronomic improvement [1]. Consistent with the main function of BRs in promoting cell elongation, mutants deficient in brassinosteroid biosynthesis and signaling display various levels of dwarfism [1]. BR biosynthesis and signaling pathways are well established and several mutants from these pathways are characterized in Arabidopsis, pea, tomato, and rice [4], [5], [6], [7]. However, relatively little is known about the specific functional role of brassinosteroids in maize [1].

More than 10 classical dwarf mutants have been described and mapped in maize [8] including four mutants involved in GA biosynthesis and signaling (*an1*, *d8*, *d9*, and *Dwarf3*) for which the underlying genes have been identified [9], [10], [11]. Only one mutant allele for a gene in the brassinosteroid synthesis pathway was recently characterized [12]. Classical dwarf mutant plants *nana plant* 1 carry a loss-of-function mutation in a DET2 homolog – a gene in the BR biosynthesis pathway. Recently, two maize genes involved in brassinosteroid synthesis were identified based on homology to Arabidopsis genes [13], [14]. *Zmdwf4*, homologous to Arabidopsis *dwf4*, was shown to rescue Arabidopsis *dwf4* mutants, in transgenic experiments [13]. RNA interference experiments with *ZmDWF1*, a homolog of Arabidopsis *DWF1*, showed that maize plants deficient in *ZmDWF1* exhibited severe dwarfism due to aberrations in stem elongation, a phenotype characteristic to mutants defective in brassinosteroid biosynthesis [14]. However, no stable maize mutants in genes involved in brassinosteroid biosynthesis and signaling have been identified and characterized.

One of the genes involved in the last steps of brassinosteroid biosynthesis encodes brassinosteroid C-6 oxidase (brC-6 oxidase). This gene has been cloned and corresponding mutants have been characterized from tomato [15], pea [16], and rice [17], [18]. The Arabidopsis genome contains two different brC-6 oxidases and only a double mutant displays the characteristic dwarf phenotype [19]. BrC-6 oxidases belong to a large family of cytochrome P450 proteins that include multiple proteins from brassinosteroid and gibberellins biosynthesis pathways [20]. Mutant plants with inactive brC-6 oxidases exhibit severe dwarfism due to almost non-existent internodes, aberrations in both leaf sheaths and blades, and a complete absence of etiolation response [15], [16], [17], [18], [19].

Here, we report the characterization of the maize mutant defective in brassinosteroid biosynthesis and describe the maize *brd1* gene that encodes brC-6 oxidase, an enzyme involved in brassinosteroid biosynthesis.

## Results and Discussion

### Cloning and characterization of the *brd1* gene in maize

**Figure 1:**
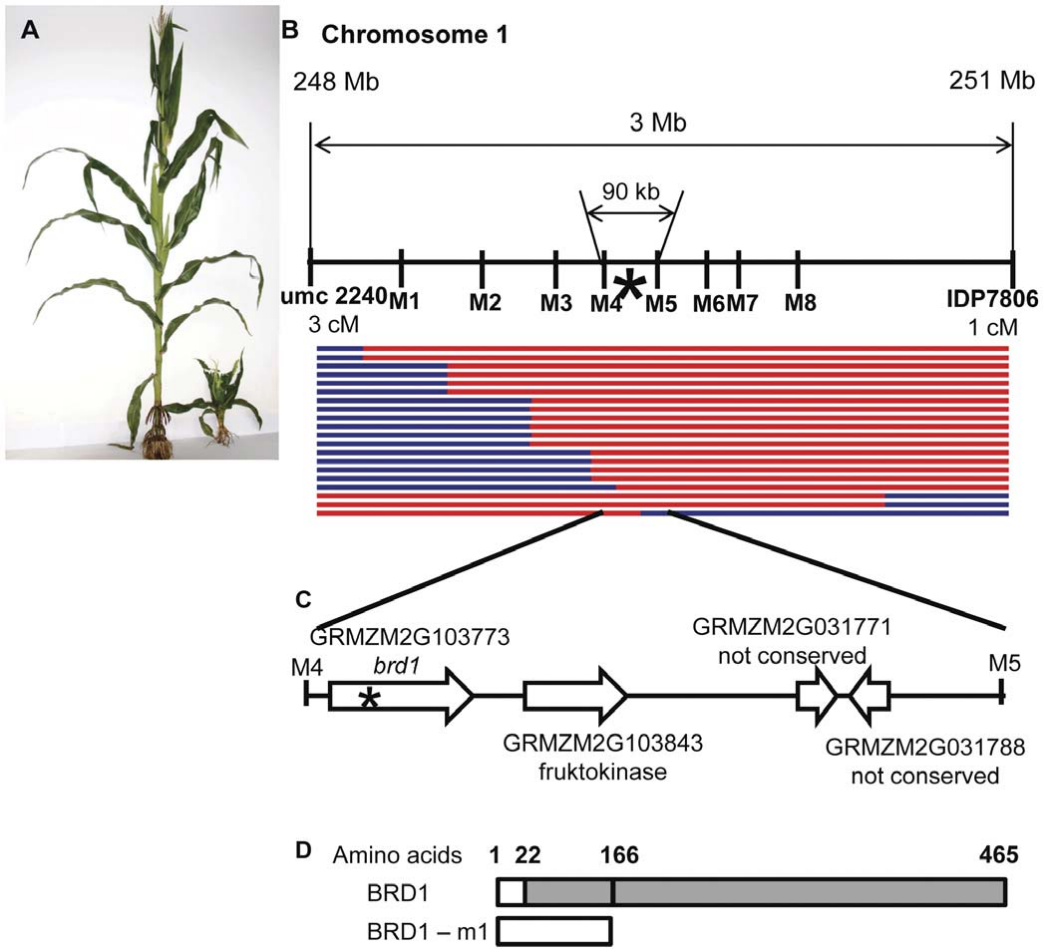
Mapping the maize *brd1-m1* mutant. **A** Comparison of a gross morphology of a severe dwarf mutant *brd1-m1* (on the right) and a wild type maize plant (on the left). **B** Physical location of the mapped gene relative to the molecular markers used for mapping. M1 through M8 are markers tightly linked to the gene (Table 1). Approximate genetic map distances between the outside markers and the mutation are shown. The star designates the location of the mutation in *brd1-m1* mapped between markers M4 and M5. Each line in the lower panel designates a particular recombinant event, where Mo17 alleles are shown in blue and B73 alleles are shown in red. **C** Predicted genes in the region between markers M4 and M5. The star indicates the location of the only mutation found in the coding regions of these predicted genes. **D** The predicted protein sequence encoded by maize *brd1* (GRMZM2G103773) gene. A nonsense mutation found in *brd1-m1* leads to the synthesis of a truncated protein of only 165 amino acids long. A cyp450 domain is shown in gray.

EMS pollen treatment was used to create a mutagenized population in the B73 genetic background [21]. During the process of creating this population, a number of mutants with interesting morphological abnormalities were noted. Plants from several of these families were crossed to Mo17 in order to generate mapping populations and the gross chromosomal position was determined through quantitative SNP assays on bulk-segregant pools [22]. One of the mutants, NM4089, exhibited a severe dwarf (Figure 1A, Figure S1) phenotype with gross alterations to leaf structure and to reproductive structures. NM4089 mutant plants could be distinguished from their wild type siblings as early as 10 days after planting and often exhibited virtually no internode elongation throughout growth and development. This mutation was mapped to maize chromosome 1 bin 10 by high-throughput bulk segregant analysis based on a Sequenom SNP-typing approach [22]. Since the location of this mutant did not correspond to the position of any known maize gene with similar phenotypic effects, we pursued a positional cloning approach to identify the allele responsible for this severe dwarf phenotype.

**Figure 2:**
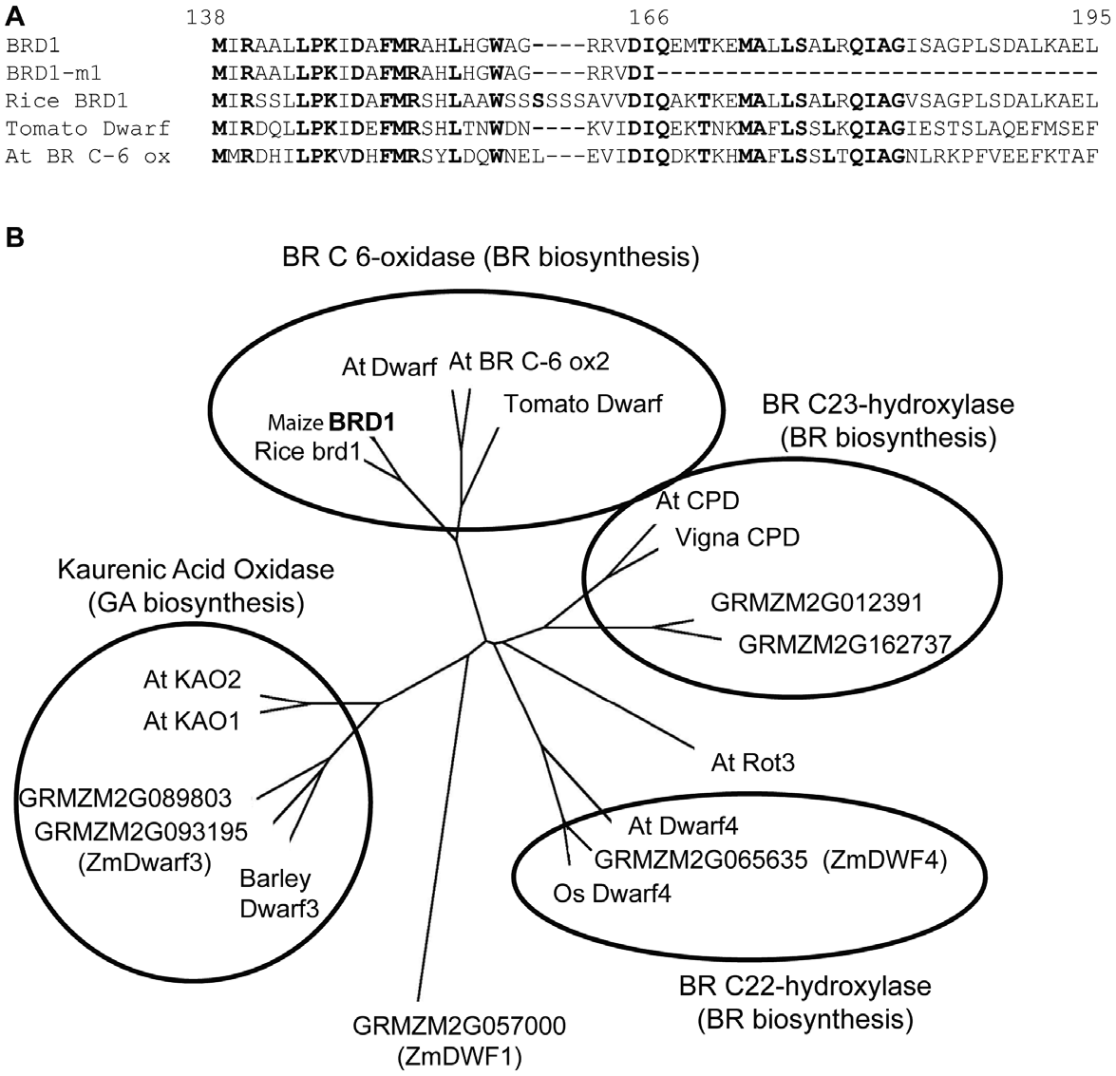
Maize *brd1* gene encodes a cytochrome P450 protein. **A** Alignment of a portion of maize BRD1 with brC-6 oxidases from other plants. **B** Phylogenetic relationships between a maize BRD1protein and other P450 proteins in maize and other plants. An unrooted tree was constructed using the neighbor-joining method. Accession numbers are as follows: rice brd1 (AB084385), OsDwarf4 (Q5CCK3), At Dwarf (AB035868), AtBRC-6 ox2 (NP_566852), Tomato Dwarf (U54770), At CPD (X87367), At Rot3 (AB008097), At Dwarf4 (AF044216), ZmDWF1 (AAS90832), ZmDwarf3 (U32579), barley dwarf3 (AF326277), At KAO1 (AF318500), At KAO2 (AF318501), ZmDWF4 (GRMZM2G065635), CPD Vigna (AF279252).

To determine the precise location of the mutation, 509 mutant plants were screened using insertion deletion polymorphism markers (IDP) [23] from bin 10 of chromosome 1 and localized the mutation to the 3 Mb region between markers umc2240 and IDP7806 (Figure 1B, Table 1). Further screening of recombinant individuals using CAPS assays or direct sequencing of B73 - Mo17 SNPs allowed mapping the mutation to a 90 kB region between markers M4 and M5 (Figure 1B, Table 1). This region contains 4 predicted genes from the filtered gene set [24] (Figure 1C) and has 1 remaining recombination. Screening SNPs located closer to the center of the 90 kB region produced no recombinants relative to the mutant phenotype. Two of the predicted genes code for short non-conserved proteins, one gene encodes fruktokinase, and the fourth gene (GRMZM2G103773) encodes brassinosteroid C-6 oxidase. The exons from all four genes were sequenced in two homozygous mutant individuals revealing a nonsense mutation in the GRMZM2G103773 gene and no mutations in any other exons within this region. This gene encodes a 465 amino acid protein. The single base pair mutation in event NM4089 resulted in the creation of a stop codon after amino acid 165 (Figures 1D, 2A). The resulting protein lacks the whole cyp450 domain and is expected to be non-functional. Due to homology with a previously characterized brassinosteroid-deficient dwarf gene in rice, the GRMZM2G103773 gene was termed *brd1 (brassinosteroid-deficient dwarf1)* with the mutant allele described as *brd1-m1*. The predicted amino acid sequence encoded by maize *brd1* is highly similar to the previously reported proteins encoded by the *Dwarf* gene in tomato (64% identity, 80% similarity) and *brd1* gene in rice (85% identity, 92% similarity), and brC-6 oxidase I in Arabidopsis (58% identity, 76% similarity) over the majority of the coding sequence (Figure 2A). These proteins encode brC-6 oxidase, an enzyme that catalyzes the last steps of brassinosteroid biosynthesis and belong to the family of cytochromes P450. This protein family includes numerous proteins that are common among higher plants and exhibit high structural similarity. Similar to rice [17], [18], the maize genome contains one gene coding for brC-6 oxidase, while dicots pea [25] and Arabidopsis [19]) appear to have two different brC-6 oxidase genes. The maize BRD1 protein sequence was compared to other P450 proteins, to determine whether it can be classified into the brC-6 ox group (cyp85 group) or other P450s groups (Figure 2B). The phylogenetic relationships inferred from protein sequences by neighbor-joining algorithm suggested that the maize protein encoded by *brd1* is more closely related to the Arabidopsis, rice, and tomato BrC-6 ox proteins than to other P450 genes, and it was designated CYP85A1 according to the nomenclature of CYP450 superfamily [20].

**Table 1:** Molecular markers used to map *brd1* gene in maize.

Marker	Position	Marker Type	Method of Detection	# recombinants
umc2240	248,125,605	IDP	PCR	17
M1	248,552,853	SNP	Sequencing	15
M2	248,854,059	SNP	HaeIII digest	11
M3	249,179,126	Present in M only	PCR	5
M4	249,308,623	SNP	HaeIII digest	1
M5	249,400,095	SNP	Sequencing	1
M6	249,501,841	SNP	Sequencing	1
M7	249,537,499	Present in M only	PCR	1
M8	249,895,322	SNP	Sequencing	1
IDP7806	251,157,750	IDP	PCR	3

### The phenotype caused by a mutation in maize *brd1* is partially rescued by BR treatment

**Figure 3:**
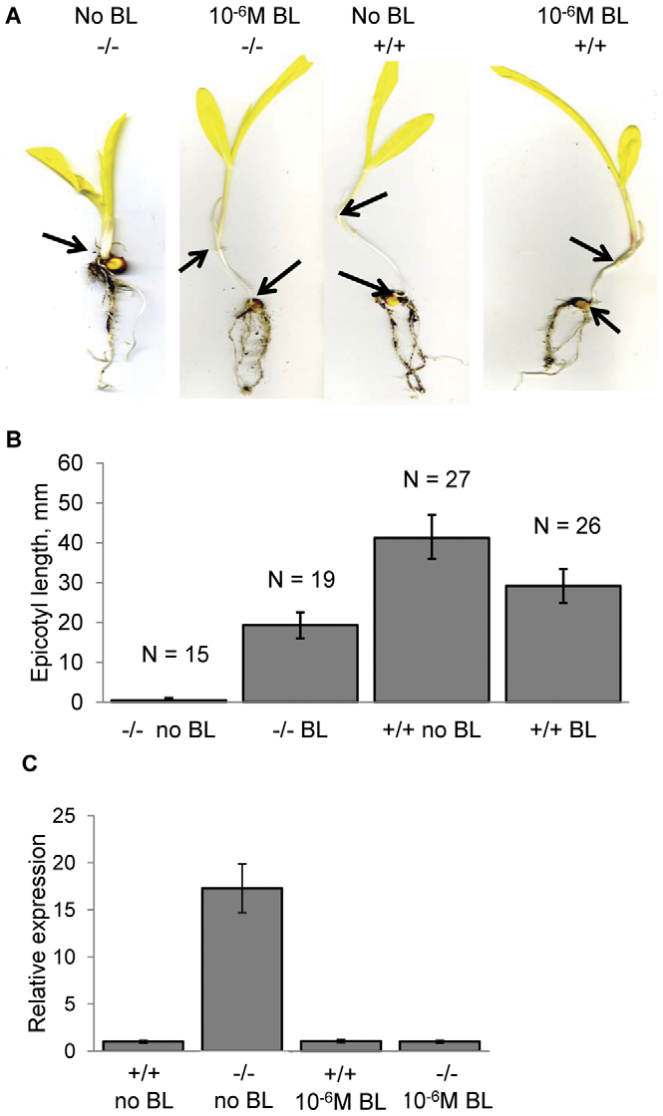
Supplementing the growth media with 10^−6^ M brassinolide (BL) partially rescues the *brd1 –m1* phenotype in maize. Plants were germinated and grown in the darkness on MS medium with or without 10^−6^ M brassinolide. At 12 days after planting the seedlings were genotyped and wild type (+/+) and mutant (−/−) plants were identified. **A** *brd1-m1* plants grown on media supplemented with brassinolide show epicotyl elongation, while mutant plants grown without brassinolide fail to show etiolation response. Arrows indicate the positions of the internodes. **B** Epicotyl length was measured in 12 day old seedlings germinated and grown in the darkness. The results are presented as mean values +/− standard deviation from four to eight plants. All of the groups exhibit statistically significant differences at p<0.01 or less (t-test). Number of plants in each category is shown above each of the columns. **C** qRT-PCR analysis of the *brd1* expression level. The expression level of *brd1* was normalized to the expression of the house-keeping *mez2* gene and shown relative to the expression of the wild type plants grown without brassinolide. No RT controls were all negative (data not shown). The difference between the *brd1* expression in mutant plants grown without brassinolide supplement and all other growth conditions is statistically significant at p<0.001 (t-test). The data are presented as mean values +/− standard deviation from three samples for each growth condition.

To confirm that the maize *brd1-m1* mutant is deficient in active brassinosteroids, *brd1-m1* and wild type siblings were grown on MS media and on MS media supplemented with 10^−6^ M brassinolide. It was difficult to grow maize plants in the agar media for long periods of time. Therefore, the effects of brassinolide treatment were evaluated for phenotypes that are manifested early in development. When grown in the dark, wild type maize plants exhibited a strong etiolation response that included epicotyl elongation, regardless of brassinolide presence in the media. In contrast, similar to rice and Arabidopsis mutants that have deficiencies in BR biosynthesis or BR sensitivity [17], [18], [19], maize *brd1-m1* plants exhibited no etiolation response, when germinated and grown in the dark, with complete absence of epicotyls or internode elongation (Figure 3A, B). This phenotype could be rescued by exogenous application of brassinolide as was shown with at least fifteen mutant plants (Figure 3A, B). A CAPS genotyping assay was used to confirm that these individuals were homozygous *brd1-m1* mutants despite the strong elongation phenotype (Figure 3). Epicotyl length in mutant plants treated with brassinolide was about 75% of that in wild type seedlings treated with brassinolide (Figure 3A, B). All four classes were significantly different from each other (t-test; p-value <0.01). Thus, supplementing with brassinolide resulted in a partial rescue of the mutant de-etiolation phenotype, similar to results observed for the *brd1* mutants in rice [17], [18].

Many of the BR biosynthetic genes, such as DWF4 and CPD, exhibit decreased transcription levels in the presence of BRs and increased transcription levels in loss-of-function BR signaling mutants, suggesting that BR biosynthesis is negatively regulated by the BRs [26], [27]. The rice *brd1* gene has been shown to be negatively regulated by active brassinosteroids, such that individuals with reduced levels of brassinosteroids exhibited elevated transcript levels of *brd1* [17]. The transcript level of *brd1* was investigated in several genotypes and treatments (Figure 3C). The *maize brd1-m1* plants exhibited substantially higher levels of *brd1* transcripts compared to wild type siblings. This increase in *brd1* transcripts in maize *brd1-m1* mutant plants could be reduced by exogenous application of brassinolide (Figure 3C). Therefore, the maize *brd1* gene is negatively regulated by brassinolide such that high levels of brassinolide decrease its transcription level. These data further suggest that maize *brd1* is indeed involved in brassinosteroid biosynthesis.

### Maize *brd1* is expressed in a variety of plant tissues

**Figure 4:**
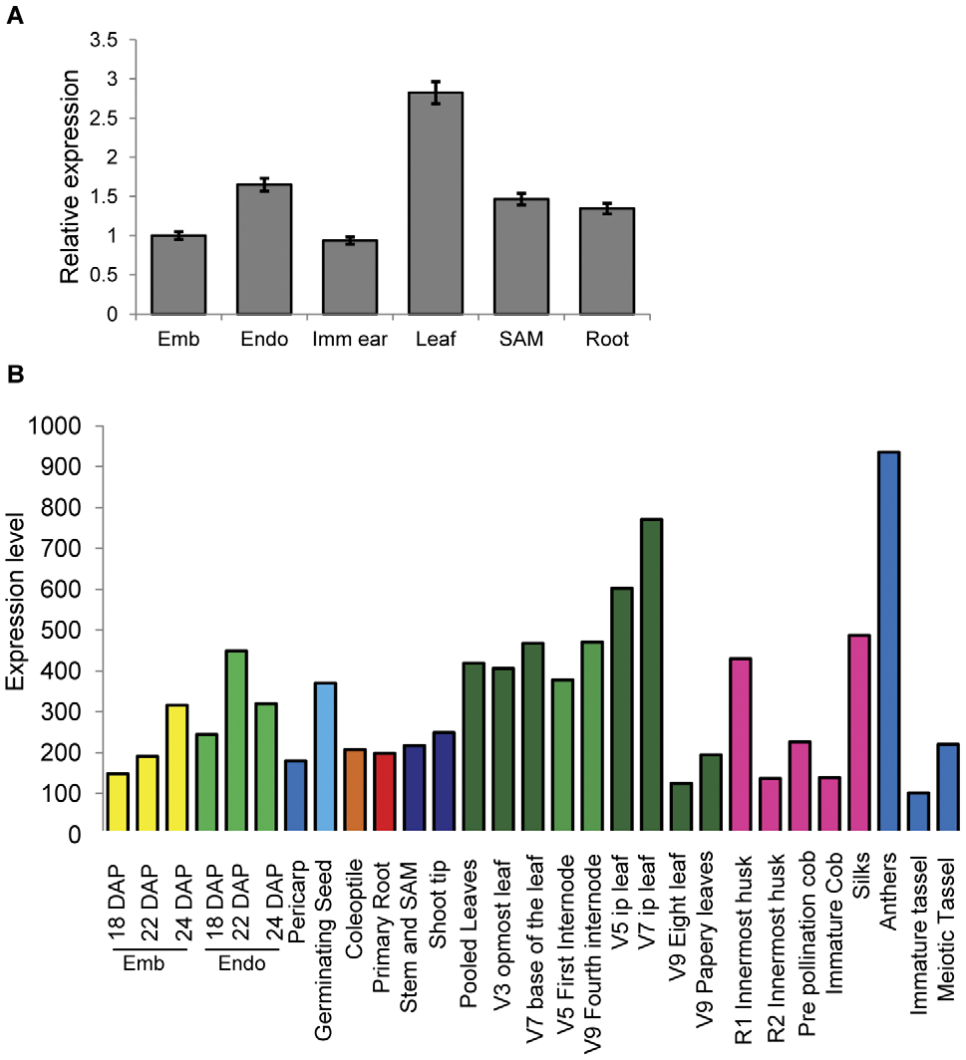
Expression pattern of maize *brd1* gene in various plant tissues. **A** Relative expression levels of maize *brd1* based on qRT-PCR analysis. cDNA was synthesized from total RNA isolated from 14 days after pollination embryo (Emb) and endosperm (endo), immature ear (Imm ear), leaf (14 day old seedling), shoot apical meristem-enriched tissue (SAM), and root (14 day old seedling) tissue. The expression level of *brd1* was normalized to the expression of the house-keeping *mez2* gene and shown relative to the expression of the wild type plants grown without brassinolide. Bars represent standard deviation values. **B** Expression levels of *Zmbrd1* in various plant tissues based on Maize Expression Atlas [28].

To further understand the location of BR biosynthesis in maize, the transcript level for *brd1* was assessed in six plant tissues using qRT-PCR (Figure 4A). The gene was detected in all tissues assessed but showed significant variation in transcript levels. The level of expression in the leaf tissue was the highest: almost 3-fold higher than in the embryo and immature ear and about 2-fold higher than in endosperm, root and shoot apical meristem-enriched tissue. The expression levels of maize *brd1* in a wider range of plant tissues were assessed using data from a maize expression atlas (Figure 4B) [28]. *Brd1* transcripts were detected in all maize tissues and at all developmental stages analyzed, with highest expression levels observed in anthers and in developing leaf tissues. Recently, it has been shown that maize primary roots are involved in production of BRs shortly after germination [29]. However, it is likely that the biosynthesis of active brassinosteroids occurs in several plant organs. The preferential expression of maize *brd1* in the leaf tissues and the severe abnormal phenotype of the leaf suggest that active brassinosteroids are synthesized in the developing leaf.

### Maize *brd1-m1* mutants exhibit severe dwarfism and other morphological abnormalities

**Figure 5:**
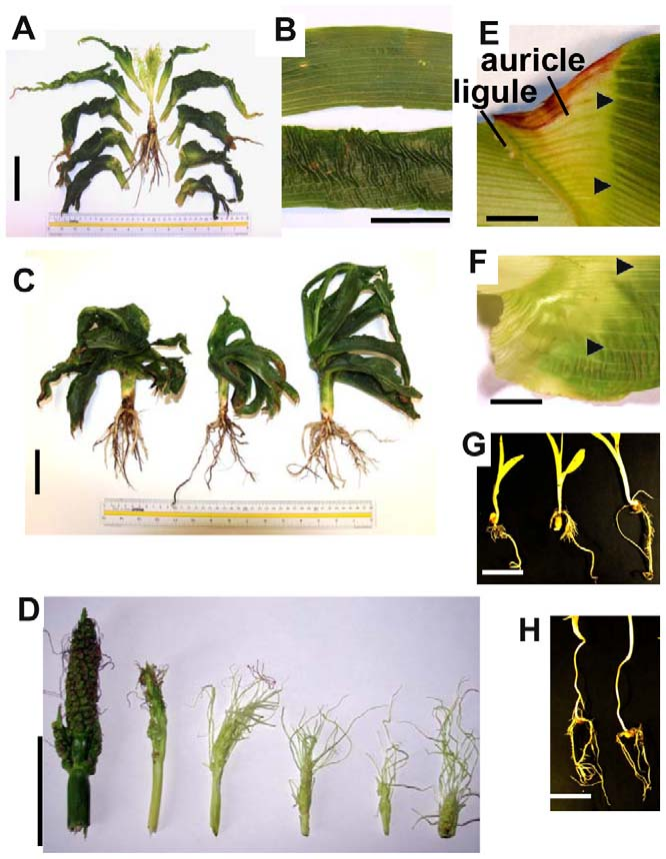
Morphological characterization of *zmbrd1-m1* mutant plants. **A** Gross morphology of *brd1-m1* mutant plants. **B** *brd1-m1* mutants exhibit severe leaf structure abnormalities. An adult leaf from the mutant plant (bottom) is compared to the adult leaf of the wild type plant (top). **C** Variability in morphology of *brd1-m1* mutant plants. **D** *brd1-m1* mutants develop feminized tassels. **E–F** *brd1-m1* mutants (**F**) exhibit abnormalities in auricle/ligular region of the leaf compared to the wild type plants (**E**). Triangle arrows indicate the border between an auricle and a blade. Adult leaves of the *brd1-m1* mutant plants lack a border between an auricle and a blade (**F**). **G–H** Root growth in *brd1-m1* mutants (**G**) is significantly impaired with almost no lateral root growth present. Three *brd1-m1* mutant plants (**G**) are compared to two wild type seedlings (**H**). Seedlings segregating for *brd1-m1* were germinated and grown in the darkness for 14 days. Scale bars on all of the pictures are 5 cm.

The gross morphology of the mutant maize *brd1-m1* plants is shown in Figure 5A and Figure S1. Individuals that are homozygous for the *brd1-m1* mutation exhibited a severe but somewhat variable dwarf phenotype. Mature *brd1-m1* mutant maize plants reached only about 20 cm in height and oftentimes were even shorter (compared to >200 cm for wild type maize plants) (Figure 5A, B). The total hieght of 40 *brd1-m1* mutant plants measured in late July (when wild-type silblings were shedding pollen) varied between 7 and 39 cm (average 19 cm; standard deviation 7 cm). *brd1-m1* mutant plants failed to form viable reproductive organs and have not demonstrated fertility in field grown conditions. Strongly feminized inflorescences of variable size were observed at the top of the plant (Figure 5A, D). These feminized tassels ranged from 2 to 10 cm in length. Silks from the tassel were commonly observed; however, we have never observed tassels that have mature anthers or pollen. The feminization of tassels is difficult to quantify as many of these plants are twisted and rolled up and a substantial proportion of the *brd1-m1* mutant plants do not form observable reproductive structures. There was essentially no internode elongation in the maize *brd1-m1* mutant plants, resulting in all leaves emerging from same location on the stem (Figure 5A). The *brd1-m1* mutant plants exhibited the normal distichous alternate phyllotaxis and normal number of leaves and continued to form new leaves throughout the whole season, indicating that leaf initiation occurred normally at the shoot apical meristem. Many aspects of the phenotypic aberrations exhibited by maize *brd1-m1* plants are similar to those observed in rice *brd1* mutants [17], [18]. Interestingly, feminized tassels were also observed in *nana plant 1* (*na1*) maize mutant plants [12] suggesting that brassinosteroids are involved in regulating tassel development.

The leaves of maize *brd1-m1* plants exhibited unusual morphology with the leaf blade tissue frequently displaying “wave” patterns (Figure 5B). Leaf sheath and ligule appeared to be smaller than in wild type plants. However, the sheath tissue is quite variable in *brd1-m1* mutants plants and is difficult to quantify due to severe twisted phenotype of these plants. The auricle tissue of the mutant leaves seemed to be enlarged (Figure 5E, F) and failed to form a distinct border between the auricle and blade of the leaf. These observations indicate that normal BR biosynthesis is critical for normal differentiation of leaf tissues. The defect in BR biosynthesis also affected the formation and development of other organs. For example, root elongation was severely inhibited in mutant plants (Figure 5G, H). The length of the main root in 14 day old *brd1-m1* mutant seedlings (n = 14) was 10 cm on average, ranging from 5 to 14 cm (standard deviation 3 cm), while the length of the main root in 14 day old wild type seedlings (n = 30) varied between 19 and 33 cm (average 26 cm, standard deviation 5 cm). These differences were statistically significant at p<0.001 (t-test). The number of “crown” roots was also severely reduced in *brd1-m1* mutant plants (3–6 in wild type 14 day old seedlings and 1–2 in *brd1-m1* homozygous plants).

**Figure 6:**
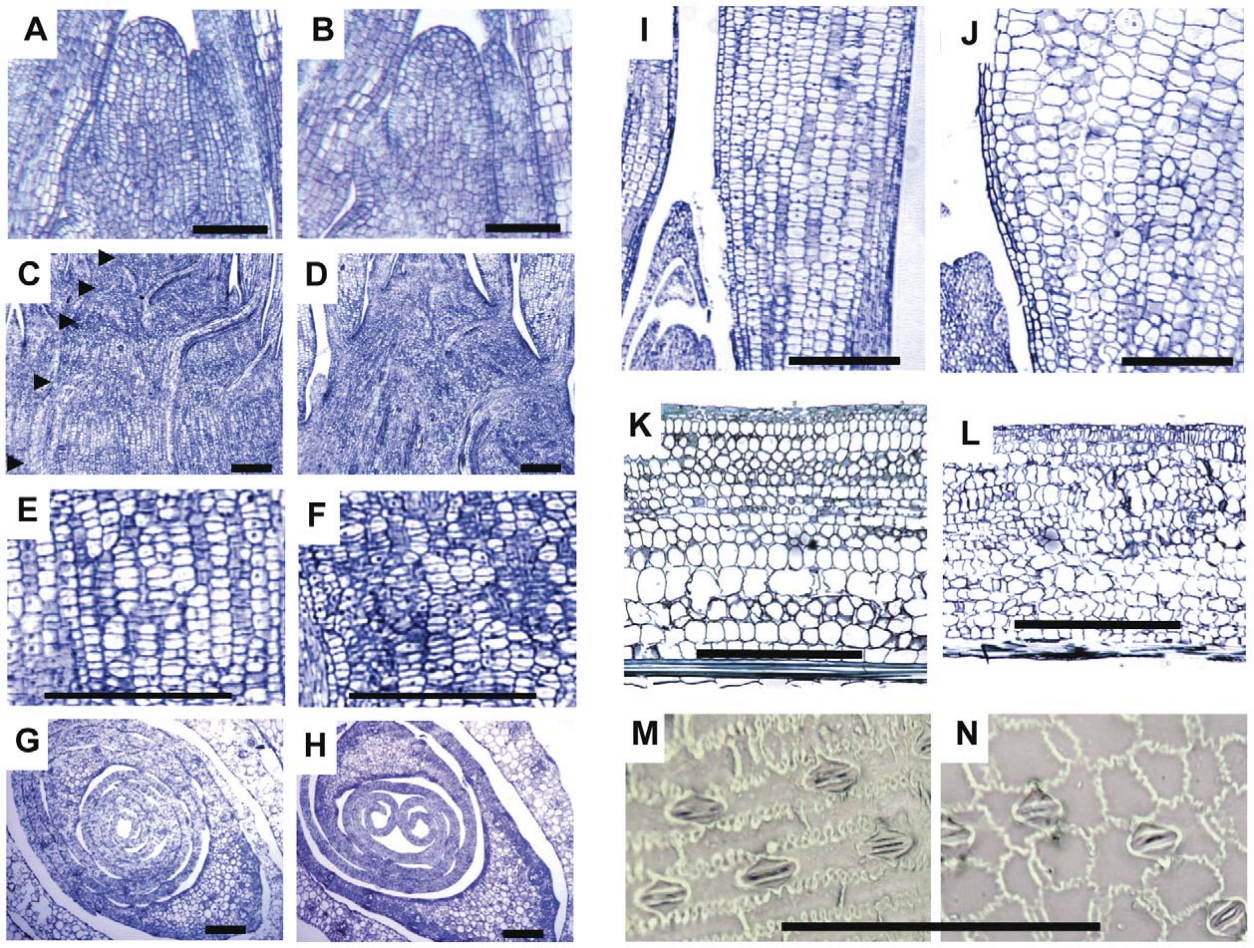
Microscopy of *brd1-m1* mutant plants. **A–F** Median longitudinal histological sections of juvenile (14-day-old) wild-type B73 (**A, C, E**) and *brd1-m1* (**B, D, F**) plants. **A–B** Shoot apical meristem. **C–F** Cells in a stem underneath shoot apical meristem. Nodes separating divisional zones are designated by arrowheads in **C. E–F** Close-up of cell files in divisional zones shown in **C–D**. **G–H** Transverse sections of wild-type (**G**) and *brd1-m1* (**H**) plants above meristem showing leaf growth. **I–J** Close-up of wild-type (**I**) and *brd1-m1* (**J**) longitudinal sections of juvenile leaves. **K–L** Adult longitudinal sheath sections in wild-type (**K**) and *brd1-m1* (**L**). **M–N** Adaxial leaf blade epidermal surface of wild-type (**M**) and *brd1-m1* (**N**). Scale bars are 100 um.

The cellular structure and developmental morphology were examined histologically in both juvenile (two-week old) and adult tissues in *brd1-m1* and wild type maize plants. The shoot apical meristems (SAM) showed no significant differences between wild-type and mutant plants (Figure 6A, B). Cells directly under the SAM lacked distinct nodal regions demarcated by divisional files of cells between nodes (Figure 6C–F), similar to the observations reported in rice *brd1* mutants [17]. However, the aberrant vasculature morphology seen in rice was not observed. The abnormal cell files present in the divisional zones beneath the SAM were also evident in longitudinal sections of juvenile (Figure 6I, J) and adult leaf sheath tissues (Figure 6K, L).

In *brd1-m1*mutant maize plants, leaves were initiated at normal intervals (Figure 6D) but frequently failed to properly wrap around the stem as they developed (Figure 6G, H). In addition, although mutant plants maintained distichous alternate phyllotaxis, transverse sections of juvenile leaves revealed rotation in the plane of the midrib (Figure 6G, H), giving seedlings a slightly twisted appearance. Mutant plants also displayed diminished sheath thickness (Figure 6K, L). Disrupted epidermal cell organization and reduced elongation were apparent in imprints of adult adaxial leaf blade surfaces (Figure 6M, N). Although reduced sheath thickness and epidermal cell layer defects were similar to those of rice *brd1* mutants [17], [18], leaf wrapping and plant rotation abnormalities displayed by *brd1-m1* mutants have not been previously reported.

Developmental abnormalities exhibited by maize *brd1-m1* mutants are generally similar to mutant phenotypes described for brC-6 oxidase homologs in rice. Inhibition of cell elongation observed in maize *brd1-m1* plants is characteristic to Arabidopsis and rice mutants for *dwf4*, *cpd*, and other genes involved in BR biosynthesis pathways [1]. Moreover, similar phenotypic effects have been observed in maize plants with reduced expression of ZmDWF1, a maize homolog of DIM1/DWF1 [14]. The disruption of normal differentiation of leaf tissues, specifically the disruption of the blade-auricle border was not previously observed in other species.

### Conclusions

This study describes isolation, positional cloning, and characterization of a BR-deficient mutant in maize. Maize *brd1* is a homolog encoding brC-6 oxidase, an enzyme that controls the last steps of brassinosteroid biosynthesis. Several lines of evidence suggest that the observed dwarf phenotype in event NM4089 is indeed caused by a mutation in GRMZM2G103773 (*brd1*), a maize homolog for brC-6 oxidase that is involved in the BR biosynthesis. First, this gene is one of only four predicted genes located in the 90 kb region, to which the mutation was localized by positional cloning. Second, GRMZM2G103773 is the only one of these four genes that contains a mutation in its coding region. Third, maize BRD1 encoded by GRMZM2G103773 is homologous to brC-6 oxidases from other plant species. Moreover, homozygous *brd1-m1* maize plants exhibit a severe dwarf phenotype, characteristic of brC-6 oxidase mutants in other species. Fourth, the *brd1-m1* phenotype is rescued by treatment with exogenous brassinolide. Finally, *brd1* expression in maize is negatively regulated by BR levels. Therefore, our evidence suggests that maize *brd1* encodes brC-6 oxidase and the nonsense allele *brd1-m1* represents a null allele. Consistent with the function of brassinosteroids in promoting cell elongation, *brd1-m1* maize mutants exhibit severe dwarfism and leaf structure aberrations at the whole leaf and cellular levels. The phenotypic abnormalities of maize *brd1-m1* mutants are similar to those described for mutants in *brd1* homologs in Arabidopsis and rice suggesting that monocots and dicots have similar BR biosynthetic pathways. Isolation of a maize mutant defective in brassinosteroid synthesis will be important for investigating the role of brassinosteroids in this important crop system.

## Materials and Methods

### Developing mapping population and gene mapping

Plants were grown in the field of University of Minnesota Saint Paul Agricultural Experimental Station (Saint Paul, MN). Maize plants exhibiting dwarf phenotypes, including line NM4089 that expressed severe dwarfism, were selected from mutant lines created by EMS mutagenesis for a maize TILLING Project [21]. F_2_ segregating populations were generated by crossing NM4089 heterozygous plants (in a B73 genetic background) to a Mo17 inbred line and then self-pollinating the resulting F_1_ plants. Seeds segregating for the NM4089 mutation were grown using standard greenhouse conditions (1∶1 mix of autoclaved field soil and MetroMix; 16 hours light and 8 hours dark; daytime temperature of 30°C and night temperature of 22°C) and mutant plants were identified 10–12 days after planting. This mutation was initially mapped to a chromosomal bin in a high-throughput bulk segregant analysis using a Sequenom platform [22]. Subsequently, a variety of Indel polymorphisms (IDP; [23]) and SNP markers were used for fine-mapping (Table 1 and Table S1). Polymerase chain reactions were performed using Qiagen Hot Start Taq Polymerase according to manufacturer's instructions (Qiagen, CA USA). PCR products were either run on a gel to observe size polymorphism or purified by Qiagen PCR purification kit (Qiagen) for sequencing or restriction analysis. Sequencing was performed by the Biomedical Genomics Center at the University of Minnesota. Due to the nature of mutation in the maize *brd1* gene, the *brd1-m1* allele could be distinguished from wild type using a CAPS assay with a *Bfa*I restriction enzyme.

### RNA isolation, cDNA synthesis, and quantitative RT-PCR (qRT-PCR)

For maize *brd1* gene expression profiling, plant tissues were ground in liquid nitrogen and RNAs were extracted using Trizol reagent according to the manufacturer's instructions (Invitrogen Corp., Carlsbad CA) and purified using the RNeasy kit, according to the manufacturer's instructions (Qiagen Corp., Valencia, CA). The quality and quantity of all purified RNA samples were assessed using agarose gel electrophoresis and the Nanodrop spectrophotometer (Thermo Scientific, Wilmington, DE). The samples collected included the following tissues: *(i)* shoot apical meristem-enriched tissue in 14 day-old seedlings, *(ii)* leaf of 14 day-old seedlings, *(iii)* embryo (14 days after pollination), *(iv)* endosperm (14 days after pollination), *(v)* root of 14 day-old seedlings, and *(vi)* immature ear (approximately 7 cm in length) tissues. One µg of total RNA was treated with DNAse I (Qiagen, CA) and used for cDNA synthesis using Invitrogen M-MLV reverse transcriptase (Invitrogen, CA) according to manufacturer's instructions and was diluted 1∶5 for use in qRT-PCR experiments. Primers for the maize *brd1* gene (Table S1) and two control genes (*GAPC*, Gene ID 542367, and *mez2*, Gene ID 542659) were designed using Primer 3.0 software [30]. qPCR reactions were performed using SYBR Green I (Bio-Rad, CA, USA) incorporation, according to manufacturer's recommendations. For each tissue sample and each genotype, three biological replicates were performed with tissue from three individual plants pooled for each replicate. Three technical replicate qPCR reactions were performed for each of the samples. Each primer pair was tested for PCR efficiency using serial dilutions of pooled cDNA samples. PCR conditions were optimized to at least 90–95% efficiency and amplification efficiency for each primer pair was calculated. The relative expression levels in each sample were determined based on the threshold cycle (Ct) value for each PCR reaction. A Ct mean value and a standard error were obtained for three technical replicates, normalized to the expression of control genes, *GAPC*, and *mez2*, and compared between biological replicates. In all cases, Ct mean values for individual biological replicates were similar and they were combined to calculate mean Ct values for each genotype-growing condition combination. A ΔCt value (difference in number of cycles to reach a threshold) was calculated for all samples relative to the wild type plants grown without brassinolide. Fold differences (FDs) for a given primer combination were calculated as (primer efficiency) ^ΔCt^. Additional t-tests were performed using three values, each representing the average of the technical replicates for each genotype/growing condition combination to assess the statistical significance of fold changes between mutant and wild type seedlings in the presence and absence of brassinolide.

### Microscopy Analyses

For shoot apical meristem (SAM), stem, and developing leaf images, homozygous *brd1-m1* and wild type B73 plants were grown in a growth chamber under standard conditions as described above, and shoot apices were dissected from two-week old seedlings. Shoot tissue was fixed, dehydrated, and embedded according to [31]. Longitudinal and transverse 8 µm sections were stained using toluidine blue O, deparaffinized, and imaged under a light microscope. The same tissue preparation and imaging procedure was followed for field-grown adult leaf sheath tissue. Epidermal cell layer images of adult field-grown homozygous *brd1-m1* and B73 plants were obtained by light microscopy of super glue (Krazy Glue, Columbus, OH) imprints of the adaxial leaf blade surface.

### Brassinosteroid supplementation experiments

Maize seeds segregating for the *brd1-m1* mutation were germinated and grown on 0.6% agarose plus Murashige-Skoog medium with or without 10^−6^ M brassinolide (BL; Sigma, WI, USA) at 30 C in the dark. For gene expression analysis, all above ground tissues of 14-day old seedlings were ground in liquid nitrogen for RNA extraction and cDNA synthesis. qRT-PCR analysis was performed as describe above. Three biological replicates were performed with tissue from three individual plants pooled for each replicate. Three technical replicate qPCR reactions were performed for each of the samples.

## Supporting Information

Figure S1 **Variation in height of** ***brd1-m1*** **mutant plants.** Mutant plants grown in the field exhibit variation in the height from 7–15 cm to 30–40 cm. The pictures were taken in July.
(TIF)

Table S1 Primer sequences used in the study.
(XLSX)
