# Supplementary figures and images for: Brd1 Gene in Maize Encodes a Brassinosteroid C-6 Oxidase
Source: PLoS One. 2012 Jan 26;7(1):e30798. doi: 10.1371/journal.pone.0030798 (PMC3266906; doi:10.1371/journal.pone.0030798)

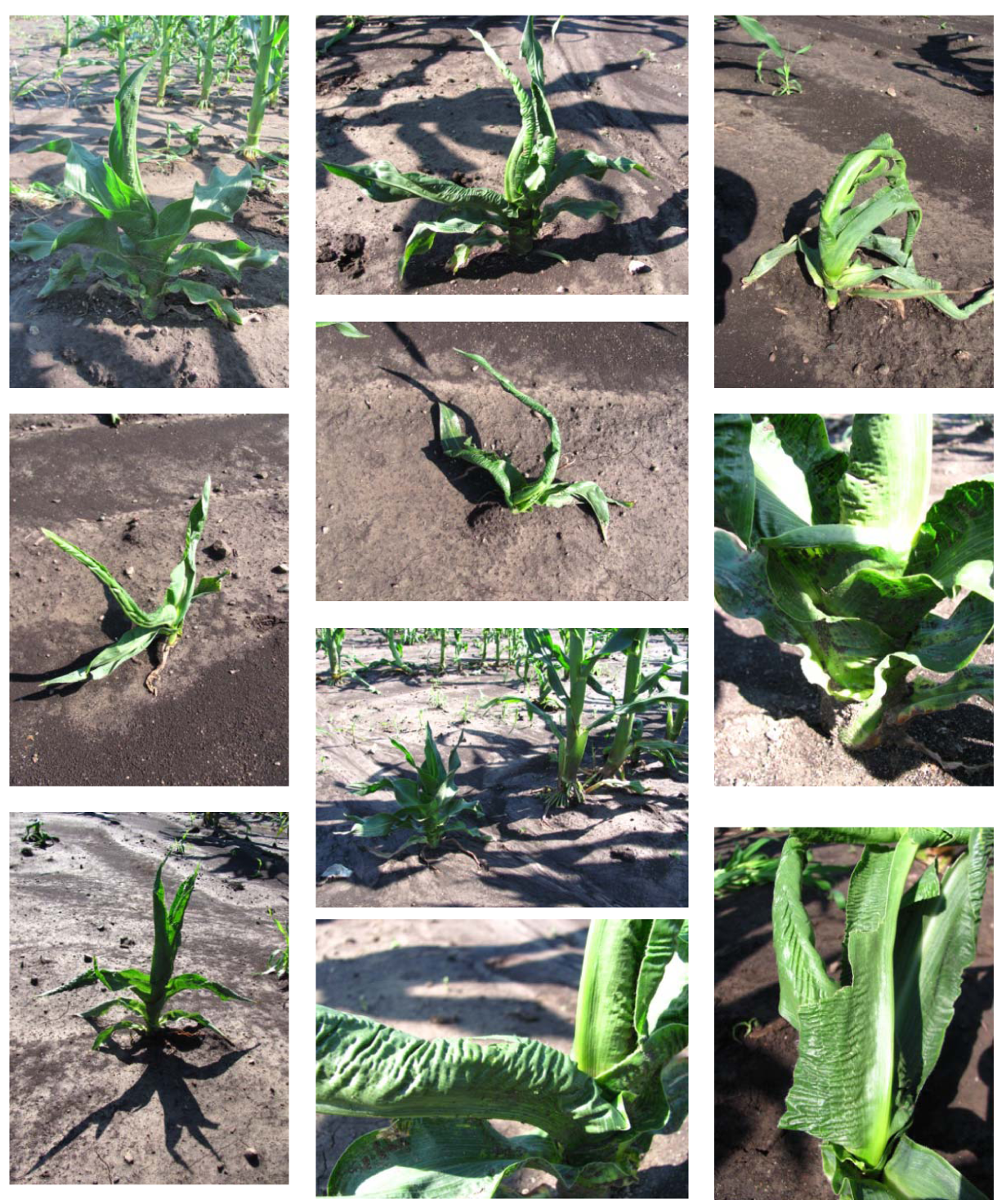

Supplement: Figure S1 — Variation in height of brd1-m1 mutant plants. Mutant plants grown in the field exhibit variation in the height from 7–15 cm to 30–40 cm. The pictures were taken in July. (TIF) [file pone.0030798.s001.tif]
